# Simulation-based inference advances water quality mapping in shallow coral reef environments

**DOI:** 10.1098/rsos.241471

**Published:** 2025-05-07

**Authors:** Pirta Palola, Varunan Theenathayalan, Cornelius Schröder, Victor Martinez-Vicente, Antoine Collin, Rosalie Wright, Melissa Ward, Eleanor Thomson, Patricia Lopez-Garcia, Eric J. Hochberg, Yadvinder Malhi, Lisa M. Wedding

**Affiliations:** ^1^ School of Geography and the Environment, University of Oxford, Oxford, UK; ^2^ Plymouth Marine Laboratory, Plymouth, UK; ^3^ Environment and Climate Change Canada, Canada Centre for Inland Waters, Burlington, Canada; ^4^ University of Tübingen, Tübingen, Germany; ^5^ Ecole Pratique des Hautes Etudes, Paris, France; ^6^ Windward Sciences, San Diego, CA, USA; ^7^ National Oceanography Centre, Southampton, UK; ^8^ Bermuda Institute of Ocean Sciences, Saint George's, Bermuda; ^9^ Environmental Change Institute, University of Oxford, Oxford, UK

**Keywords:** neural network, Bayes, remote sensing, statistical inference, coral reef, machine learning, radiative transfer, inverse problem

## Abstract

Human activities are altering coral reef ecosystems worldwide. Optical remote sensing via satellites and drones can offer novel insights into where and how coral reefs are changing. However, interpretation of the observed optical signal (remote-sensing reflectance) is an ill-posed inverse problem, as there may be multiple different combinations of water constituents, depth and benthic reflectance that result in a similar optical signal. Here, we apply a new approach, simulation-based inference, for addressing the inverse problem in marine remote sensing. The simulation-based inference algorithm combines physics-based analytical modelling with approximate Bayesian inference and machine learning. The input to the algorithm is remote-sensing reflectance, and the output is the likely range (posterior probability density) of phytoplankton and suspended minerals concentrations, coloured dissolved organic matter absorption, wind speed and depth. We compare inference models trained with simulated hyperspectral or multispectral reflectance spectra characterized by different signal-to-noise ratios. We apply the inference model to *in situ* radiometric data (*n* = 4) and multispectral drone imagery collected on the Tetiaroa atoll (South Pacific). We show that water constituent concentrations can be estimated from hyperspectral and multispectral remote-sensing reflectance in optically shallow environments, assuming a single benthic cover. Future developments should consider spectral mixing of multiple benthic cover types.

## 1. Introduction

Coral reefs support unique biodiversity and hold immense cultural and economic significance for coastal communities around the world [1,2]. Alarmingly, coral reef ecosystems are undergoing rapid changes in community composition and ecological functions due to a combination of global and local drivers of change [3,4]. Water quality is one of the most important determinants of coral health and resilience at a local scale [5–7]. Concentrations of phytoplankton, minerals and coloured dissolved organic matter (CDOM) are essential optically active water quality parameters that can inform us about ecological and biogeochemical processes taking place in the reef system [8]. However, little is known about the spatial distribution and temporal variability of these water quality parameters within and across different coral reef systems [1,9,10]. Indeed, understanding the spatio-temporal dynamics of water quality is a key knowledge gap in coral reef science and management [10–12]. Field-based water quality surveys tend to have limited spatial coverage, and long-term monitoring studies are rare [9,13,14].

Remote sensing via satellites, aeroplanes or drones is a powerful tool for mapping and monitoring coral reef environments over large geographical regions [11,15]. Marine remote sensing is based on estimating ecological parameters of interest from remote-sensing reflectance $R_{rs}$ [16]. However, estimating water constituent concentrations from remotely sensed data is challenging in optically shallow coastal environments, where benthic reflectance makes a major contribution to the total optical signal [17,18]. Indeed, reliably mapping water constituents in optically shallow waters remains one of the major unresolved challenges of marine remote sensing [8,19].

Shallow water remote-sensing methods can be divided into empirical and analytical approaches [17]. Traditional empirical methods use statistical regression analysis to define the relationship between $R_{rs}$ and *in situ* measurements [20,21]. Empirical methods are computationally simple and thereby generally easy to implement [20]. However, empirical methods usually suffer from limited transferability [17,22], i.e. a decreased performance beyond the local study site and in changing environmental conditions [19,23,24]. Analytical methods, in contrast, offer greater potential for transferability to different locations and applicability in environments characterized by variable water constituent concentrations [17,25]. Analytical methods leverage the radiative transfer equation that mathematically describes the transfer of electromagnetic radiation in the aquatic medium [16,26]. In practice, solving the radiative transfer equation requires the use of empirical approximations, and hence, these methods are commonly known as *semi*-analytical methods [27–30]. Semi-analytical methods require high-quality bio-optical data for model calibration and are relatively computationally expensive [17,24,25,31].

**Figure 1.**
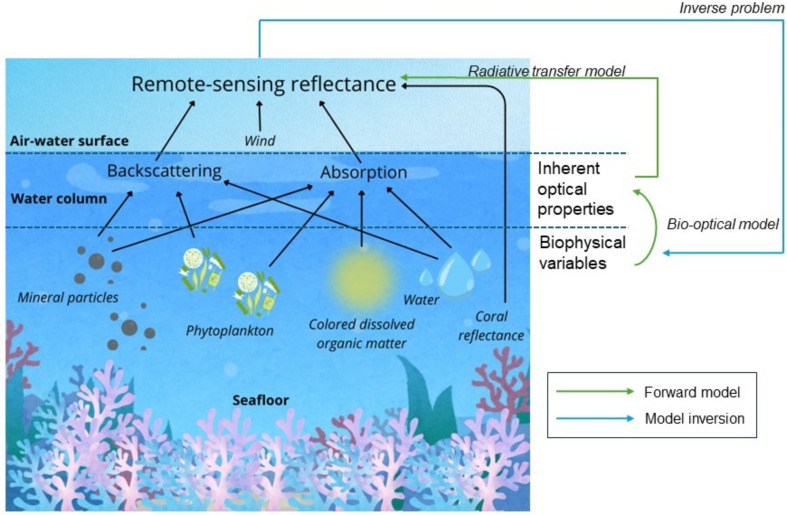
The inverse problem of marine remote sensing. Light travelling through the water column is absorbed and scattered by optically active water constituents, such as suspended minerals, phytoplankton and coloured dissolved organic matter (CDOM). The impacts of these biophysical variables on light transfer can be described through the bio-optical modelling of inherent optical properties, i.e. the backscattering and absorption coefficients associated with the water constituents. Additionally, in optically shallow environments, the signal measured by the optical sensor (remote-sensing reflectance) is affected by reflectance from the seafloor. Remote-sensing reflectance just above the water column can be modelled from the inherent optical properties and benthic reflectance using radiative transfer modelling.

To find a solution, semi-analytical methods must solve an inverse problem: the input parameters of the radiative transfer and bio-optical models must be inferred from the output of the model (figure 1) [32]. Spectral optimization methods iteratively minimize the distance between modelled ${\hat{R}}_{rs}$ and observed $R_{rs}$. The distance between the two spectra is estimated by minimizing a cost function, such as least squares error [28,33]. Semi-analytical inversion methods leveraging hyperspectral data can be used to simultaneously estimate bathymetry, benthic cover and water column optical properties [34]. However, the inversion problem of coastal marine remote sensing is mathematically ill-posed because the solution is not unique [35,36]. In other words, there may be multiple different combinations of water constituents, depth and benthic reflectance that result in a similar optical signal [34,37]. Yet, traditional semi-analytical inversion methods only provide a single solution without an estimate of uncertainty in the result [28,38]. Spectral optimization methods may fail to retrieve the global minimum and instead provide the local minimum near a given initial guess as the ‘best’ solution [33,38]. Furthermore, the performance of spectral optimization methods is highly dependent upon the quality of the input spectra, as well as the choice of starting values [39,40].

In this study, we investigate the extent to which probabilistic machine learning can be leveraged to map water constituent concentrations in coral reef environments from hyperspectral and multispectral data. We apply a simulation-based inference (SBI) algorithm that tackles the inverse problem of marine remote sensing in optically shallow coral reef waters. Rather than providing an estimate of a single ‘best’ solution to the inverse problem, the SBI algorithm produces a distribution of plausible solutions (a posterior probability density) of the water quality parameters, wind speed and depth. Additionally, we examine how the performance of the SBI algorithm depends on (i) the spectral resolution (hyper- vs multispectral); and (ii) the signal-to-noise ratio of the spectral data.

## 2. Methods

We apply SBI, which combines physics-based analytical modelling with approximate Bayesian statistics and machine learning. We design the algorithm specifically for optically shallow reef environments (brown coral, less than 20 m depth). The input to the algorithm is $R_{rs}$, and the output consists of posterior probabilities for phytoplankton and minerals concentrations, absorption by CDOM, wind speed and depth. In the following sections, we summarize the principles of SBI and describe the key steps of algorithm development.

### 2.1. Simulation-based inference

Radiative transfer modelling software can be used to simulate remote-sensing reflectance $R_{rs}$ and other radiometric quantities of interest under different environmental conditions [41,42]. However, an analytical expression for the likelihood function is typically not available for these computationally expensive simulators, and conventional Bayesian inference methods cannot therefore be applied [43,44]. SBI is an approximate Bayesian inference method that circumvents the problem of likelihood intractability and only requires the ability of sampling from the likelihood, which corresponds to a forward evaluation of the simulator. Different variants of SBI exist, which either target the likelihood function, a likelihood ratio or the posterior distribution directly [45]. In the presented work, we conduct neural-posterior estimation, which uses neural networks for conditional density estimation to approximate the posterior distribution [43,46].

**Figure 2.**
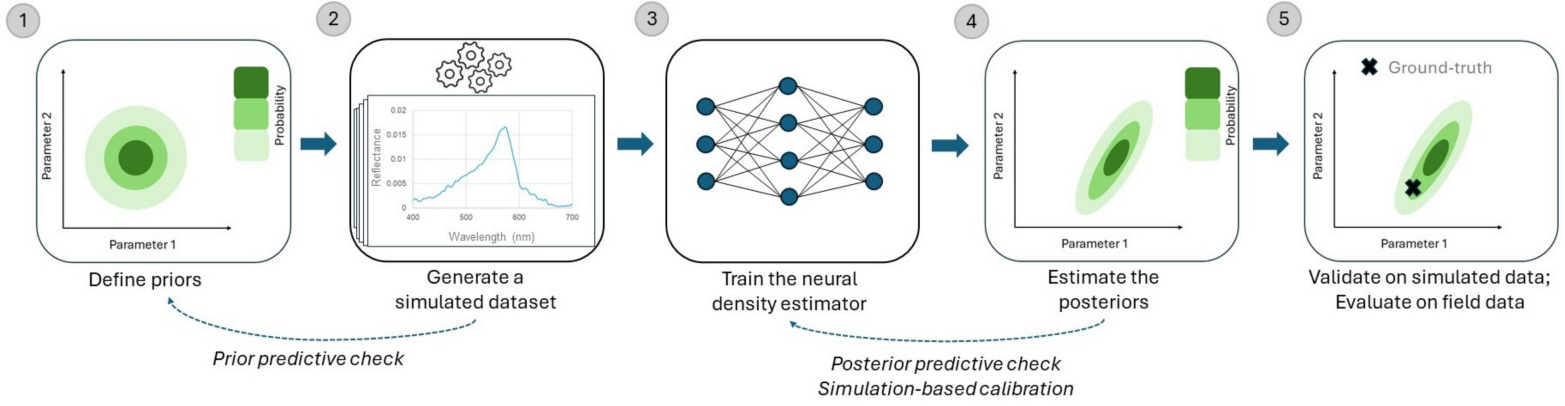
Illustration of the simulation-based inference workflow. After defining the prior distribution (1), we generate a simulated dataset (2), which is used to train a conditional density estimator (3). Once the neural network is trained, we can evaluate it at different observations to get the posterior distribution (4). Finally, the result is validated on different data modalities (5). To control the workflow, we additionally perform prior and posterior predictive checks.

The simulator, in this case a marine radiative transfer model (EcoLight, v. 5.3, Numerical Optics Ltd), takes a vector of input parameters θ (phytoplankton and minerals concentrations, absorption by CDOM, wind speed and depth) and produces an output x~p(x|θ) (${\hat{R}}_{rs}$) (figure 2). A simulated dataset is generated by drawing samples from the priors π(θ) defined independently for each parameter following a literature review (see §2.3). The simulated dataset is used to train a conditional density estimator $q_{\Phi }\left(\theta \right)$ to approximate the true posterior distribution p(θ|x). The density estimator can then be applied to real measured data $x_{o}$ to estimate the posterior distribution $p(\theta |x_{o})\propto p{(x}_{o}|\theta )\pi (\theta )$.

The density estimator applied was a mixture density network with three layers with 90 nodes each and six mixture components [47]. The density estimator is amortized, allowing for inference without having to repeat the computationally expensive simulations or to run additional Markov chain Monte Carlo sampling [43,45,46].

The inference scheme, hereafter the SBI algorithm, was coded in Python 3.10 and leverages the sbi toolbox for SBI [48]. All data and code are freely available via the Open Science Framework data repository [49]. All SBI models were trained on a High-End CPU (see electronic supplementary material, appendix A for details).

### 2.2. Simulator

EcoLight, the state-of-the-art model for solving radiative transfer equations, was used to create a large, simulated dataset [50]. The ${\hat{R}}_{rs}$ spectra were simulated between 400 and 700 nm at a spectral resolution of 5 nm (61 bands). EcoLight couples a water column bio-optical model with models of bottom reflection, air–water surface and atmosphere. The bottom reflectance was specified as brown coral. The brown coral reflectance was the average spectrum calculated from a global benthic reflectance dataset [51]. Wind speed was included in the model as it affects sea surface roughness and thereby the transfer of light across the air–sea interface (electronic supplementary material, appendix B) [16,52].

In the ocean optics literature, different terms and notations are sometimes used to refer to the same bio-optical variables [16,53–55]. To avoid confusion, it is important to make the distinction between biophysical variables (e.g. concentrations of phytoplankton and minerals) and inherent optical properties (e.g. absorption and scattering by phytoplankton and minerals). In this study, we follow the terms used in the EcoLight technical documentation (table 1) [50] and by [54]. Phytoplankton concentration (mg m^−3^) refers to the concentration of chlorophyll-bearing particles, approximated by chlorophyll-a concentration [50]. The mineral concentration is the concentration of inorganic particles, such as suspended mineral sediments, measured in g m^−3^ [50]. CDOM absorption is measured in m^−1^ at 440 nm [50]. Full details of the parametrization of the bio-optical model in EcoLight are provided in electronic supplementary material, appendix B.

**Table 1.** Input parameters to the EcoLight bio-optical model: definitions and units.

parameter	definition or synonym	unit
phytoplankton concentration	concentration of chlorophyll-bearing particles, approximated by chlorophyll-a concentration	mg m^−3^
CDOM absorption	yellow matter; gelbstoff	m^−1^ at 440 nm
minerals concentration	concentration of inorganic particles, such as suspended mineral sediments	g m^−3^
wind speed	speed of wind affecting sea surface roughness	m s^−1^
depth	depth of the water column	m

### 2.3. Priors and training data

**Figure 3.**
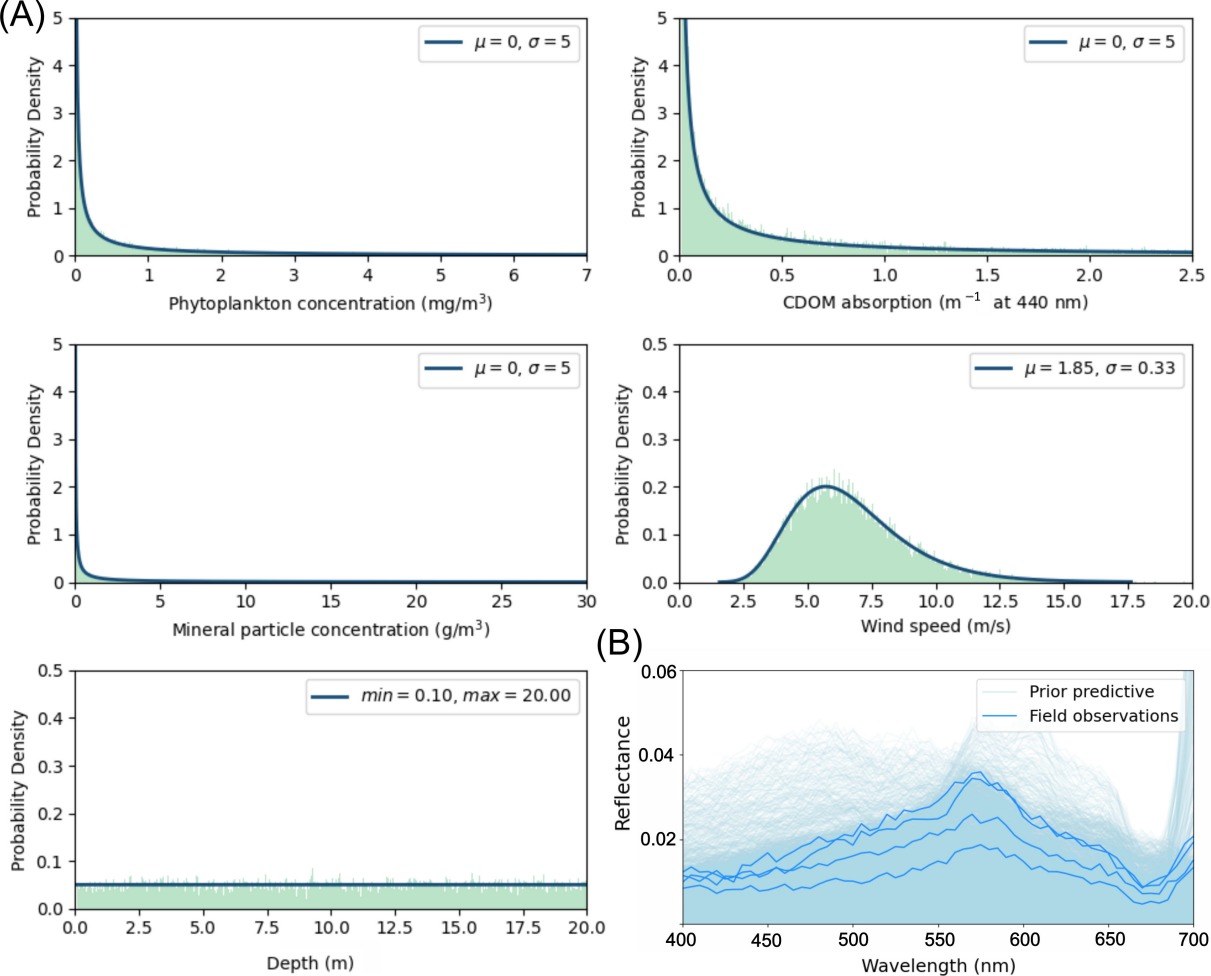
(A) Prior distributions for each of the EcoLight input parameters. A probability density function (in blue) is shown in addition to a histogram (in green) of the 30 000 samples drawn. The parameters of the lognormal distribution are the mean μ and s.d. σ. CDOM = coloured dissolved organic matter. (B) Prior predictive check. The four $R_{rs}$ spectra observed in the field (dark blue) lie within the support of the simulated ${\hat{R}}_{rs}$ spectra (light blue).

Prior distributions were defined independently for each of the five input parameters to EcoLight: wind speed, depth, absorption by CDOM and concentrations of phytoplankton and minerals (figure 3A). Lognormal distributions were used for CDOM, phytoplankton and minerals [35,56,57]. Coral reef environments are in general characterized by low concentrations of optically active water constituents (electronic supplementary material, appendix C). Occasionally, however, short-term events such as sediment plumes induced by heavy rainfall may increase the concentrations by several orders of magnitude [58]. While the mean concentration of suspended minerals is typically less than or close to 1 g m^−3^ [10,53], storm-driven increases in sediment run-off and resuspension can lead to suspended minerals concentrations of 5−30 g m^−3^ [59]. A lognormal probability distribution captures this variability by assigning a high probability to low concentrations, while also accommodating for the possibility of high concentrations [57]. The distributions were truncated to exclude unrealistically high values. The thresholds for truncation were 7 mg m^−3^ for phytoplankton concentration, 2.5 m^−1^ at 440 nm for CDOM absorption and 30 g m^−3^ for minerals concentration. The thresholds were based on a literature review (electronic supplementary material, appendix C).

The prior for wind speed was defined as a lognormal distribution using the global wind dataset provided by E.U. Copernicus Marine Service Information (https://doi.org/10.48670/moi-00182) [60] (electronic supplementary material, appendix B, figure SB.1). Lastly, a uniform distribution was assigned for the depth variable, indicating that any depth in the specified range is equally probable. As the detectability of the bottom in coral reef waters has been estimated to be up to 20 m [51], the depth range was limited to 0.10−20.00 m (figure 3A). To validate the specified prior distributions, we conducted a prior predictive check. First, we sampled from the prior and ran the simulator 1000 times. We then compared the simulated data with field observations (see §2.5) and found that all observations were within the support of the simulated data (figure 3B).

We then created a large dataset of simulated ${\hat{R}}_{rs}$ spectra and corresponding input parameters θ using EcoLight; 30 000 samples were drawn from the prior distribution and used to produce 30 000 different parametrizations of EcoLight; 29 000 simulated data points were used as training data, while 1000 simulated data points were set aside as a test dataset to conduct inference diagnostics (see §2.4). We assumed no correlation between the different variables, as such correlations may vary from system to system and with changing environmental and climate conditions [10,61].

#### 2.3.1. Signal-to-noise ratio

Stochasticity was introduced into the simulations by adding noise to the spectral data [40]. Optical remote sensing is affected by noise from multiple sources, including transmission errors, thermal effects and photon noise [62–64]. The combination of noise from different sources can be represented by a Gaussian noise term [64]. The variance of the Gaussian is calculated from a signal-to-noise ratio: $SNR=E[signal_{\lambda }^{2}]/E[noise_{\lambda }^{2}]$ and $\sigma^{2}=(\sum_{\lambda }signal_{\lambda }^{2}/N)/SNR$, where N is the number of wavelengths λ. We tested three levels of signal-to-noise ratio (50, 100 and 500) and assumed an additive noise model [64,65]: ${signal}_{observed,\lambda }={signal}_{true,\lambda }+{noise}_{\lambda }$.

Signal-to-noise ratios are wavelength-dependent and vary from sensor to sensor [62,63,66]. The aim of this study is to demonstrate a new general solution to the inverse problem of marine remote sensing. A detailed, sensor-specific characterization of the noise model was beyond the scope of this study.

#### 2.3.2. Spectral resolution

To examine the impact of spectral resolution on inference performance, we downsampled the simulated reflectance data to correspond to the bands of a multispectral drone sensor (MicaSense RedEdge-MX Dual Camera System). This camera system has seven narrow bands in the visible region: coastal blue (444 nm), blue (475 nm), green 1 (531 nm), green 2 (560 nm), red 1 (650 nm), red 2 (668 nm) and red edge (705 nm). Remote sensing via airborne drones enables a very high spatial resolution (less than 10 cm) and the possibility to capture and monitor rapidly changing water quality conditions [67]. This makes it well suited for water quality monitoring in coral reef environments [68].

### 2.4. Inference diagnostics

A test dataset of 1000 simulated data points (simulated ${\hat{R}}_{rs}$ and the corresponding input parameters θ) was used to conduct three types of inference diagnostics before applying the SBI algorithm to field-collected data. First, in a posterior predictive check, we drew a single simulated ‘ground-truth’ ${\hat{R}}_{rs}$ spectrum from the test dataset. We then created a new simulated dataset (hereafter, PPC dataset) using parameters $\theta_{posterior}$ (*n* = 1000) which were sampled from a posterior distribution estimated for the simulated ground-truth spectrum ${\hat{R}}_{rs}$ [69,70]. We then checked that the ground-truth spectrum lies within the support of the simulated ${\hat{R}}_{rs}$ spectra in the PPC dataset. Second, we conducted simulation-based calibration using the entire test dataset to assess if the variances of the posterior are balanced, in other words neither over-confident nor under-confident [71,72]. Simulation-based calibration is based on calculating a rank statistic from the marginal posterior estimates. If the posteriors have well-calibrated uncertainties, the rank statistics should be uniformly distributed [72]. An empirical cumulative distribution function of the rank statistics with respect to the 95% confidence interval of a uniform distribution can be used to visualize the simulation-based calibration [72]. Third, we quantified inference performance on the simulated test dataset by calculating coverage probability. The coverage probability measures how often the true parameter value falls within the credible intervals (between the 5th and 95th percentiles) of the posterior distributions.

### 2.5. Field data

**Figure 4.**
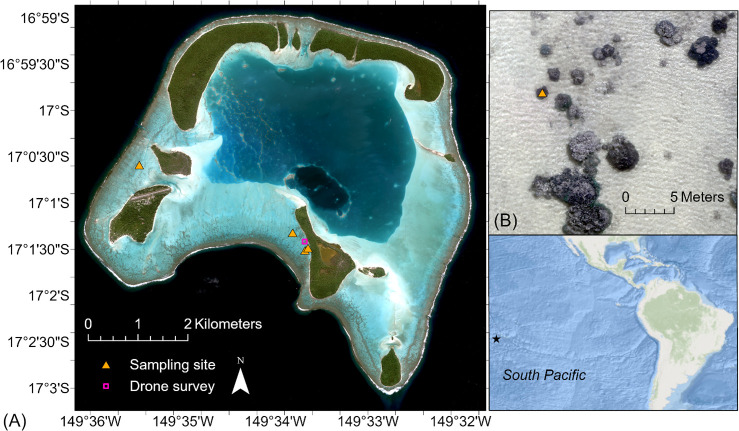
(A) Field sampling sites and drone survey location in Tetiaroa, a coral reef atoll in the South Pacific. (B) Imagery from the drone survey. Satellite imagery: Pléiades ©CNES 2022, Distribution AIRBUS DS, tous droits réservés. Usage commercial interdit. Basemap credit: Esri, Garmin, GEBCO, NOAA NGDC and other contributors.

Field data for the evaluation of the SBI algorithm performance were collected from the coral reef atoll of Tetiaroa in the South Pacific (Te Ao Māꞌohi, French Polynesia) in July and August 2022 (figure 4A). Only sampling sites from brown coral patches (*Porites lobata*) not covered in turf, sand or algae were included, leaving a dataset of four samples for the algorithm application. Additionally, SBI was applied to drone data, for which ground-truth data from one sampling site was available (figure 4B).

#### 2.5.1. Hyperspectral measurements

Just below surface ($0^{-}$), hyperspectral measurements were conducted at each sampling site using a pair of TriOS RAMSES irradiance and radiance radiometers mounted on a frame [55]. The TriOS RAMSES radiometers cover the wavelength range of 320−920 nm at a spectral resolution of 3.3 nm. The irradiance sensor was pointed straight up to measure downwelling irradiance $E_{d}(0^{-},\lambda )$, and the radiance sensor was pointed straight down to measure upwelling radiance $L_{u}(0^{-},\lambda )$. The irradiance and radiance measurements were repeated three−five times over a 2 min time interval [55]. The mean of these repeated measurements was used in the subsequent calculations. Sub-surface remote-sensing reflectance $r_{rs}(0^{-},\lambda )$ was then calculated from these measurements ${r_{rs}(0^{-},\lambda )=L}_{u}(0^{-},\lambda )/E_{d}(0^{-},\lambda )$ (sr^-1^). Sub-surface remote-sensing reflectance was converted to remote-sensing reflectance just above the water surface $R_{rs}(0^{+},\lambda )$ applying $R_{rs}=\;0.5r_{rs}/(1-1.5\;r_{rs})$ [28].

Cubic spline interpolation was used to convert the data from 3.3 nm resolution to 5 nm so as to match the spectral resolution of the simulated dataset. Additionally, to evaluate the versions of the SBI model trained on multispectral reflectance data (corresponding to the bands of the MicaSense RedEdge-MX Dual Camera System), a downsampled field reflectance dataset was created.

#### 2.5.2. Drone survey

A drone survey was conducted over a shallow reef in Tetiaroa on 3 August 2022. The data were acquired using a DJI Inspire 2 drone equipped with a MicaSense RedEdge-MX Dual Camera System. The drone was flown at an altitude of 20 m, producing less than 5 cm resolution imagery. Radiometric calibration of the imagery was performed using a MicaSense reflectance panel as the calibration target. Images of the panel were recorded before and after the flight, by holding the drone above the panel. The calibration was performed in Agisoft Metashape (v. 1.8.4), using the reflectance values given on the panel, lighting conditions recorded by the downwelling light sensor and the standard Agisoft radiometric calibration workflow. Ground-control points for georeferencing were taken using a differential global navigation satellite system (GNSS; Emlid Reach RS+). The imagery was processed in Agisoft to generate a single orthomosaic. Water sampling was conducted at a single sampling site on top of a patch of brown coral immediately after the drone survey. The surface reflectance at the sampling site was extracted from the orthomosaic using the ‘extract’ function in the Raster package in R (v. 3.6−26) [73]. The value returned by the extraction method was interpolated from the values of the four nearest pixels.

#### 2.5.3. Water sampling and ancillary data

Discrete water samples were collected using dark 1 l HDPE bottles at each sampling site and transported to the United Kingdom (UK) for laboratory analysis of chlorophyll-a concentration and CDOM absorption. The samples were collected from surface water (less than 50 cm below surface). Before sample collection, the bottles were flushed twice with water from the sampling site. Immediately after sample collection, the bottles were placed in a cool and dark box. The samples were filtered and frozen within 2−3 hours. At the local research station and during transportation to the UK, the samples were kept frozen at −20°C. Upon arrival in the UK, the samples were stored in a −80°C freezer until analysis.

We followed the protocol published by the International Ocean Colour Coordinating Group (IOCCG) for the spectrophotometric measurement of CDOM absorption [74]. Chlorophyll-a concentration was fluorometrically measured following the Environmental Protection Agency Method 445.0 [75]. Additionally, turbidity measurements were conducted *in situ* using a multiparameter water quality sonde (EasyProbe 30). Suspended particulate matter (SPM) concentration was estimated from turbidity using an empirical relationship [76]: $\left[SPM\right]=m\times Turbidity$, where m is an empirically estimated conversion factor [76]. The relationship between turbidity and SPM concentration may vary in space and time, for example due to changes in grain size of the suspended particles [76]. According to a study focused on the Great Barrier Reef, the conversion factor may vary between 1 and 5 [76]. The estimated SPM concentrations are therefore highly uncertain. To take this into account, we report the estimated range of SPM concentrations (defined by the lower (1) and upper (5) limit of conversion factors) rather than a single value. SPM consists of both organic and inorganic particles, and therefore only provides an upper boundary for mineral concentration.

Depth was measured using a weighted transect line and benthic cover was recorded using a digital camera. The site location was recorded at an accuracy of at least 50 cm using a Bad Elf Flex GNSS receiver. Wind speed was approximated by the same researcher every day.

## 3. Results

We start by presenting the inference diagnostics results. We then provide results of SBI applications to field-collected data and discuss how SBI performance is affected by signal-to-noise ratio and spectral resolution. We highlight novel insights that can be drawn from applying a probabilistic machine learning approach in marine remote sensing.

### 3.1. Inference diagnostics

**Figure 5.**
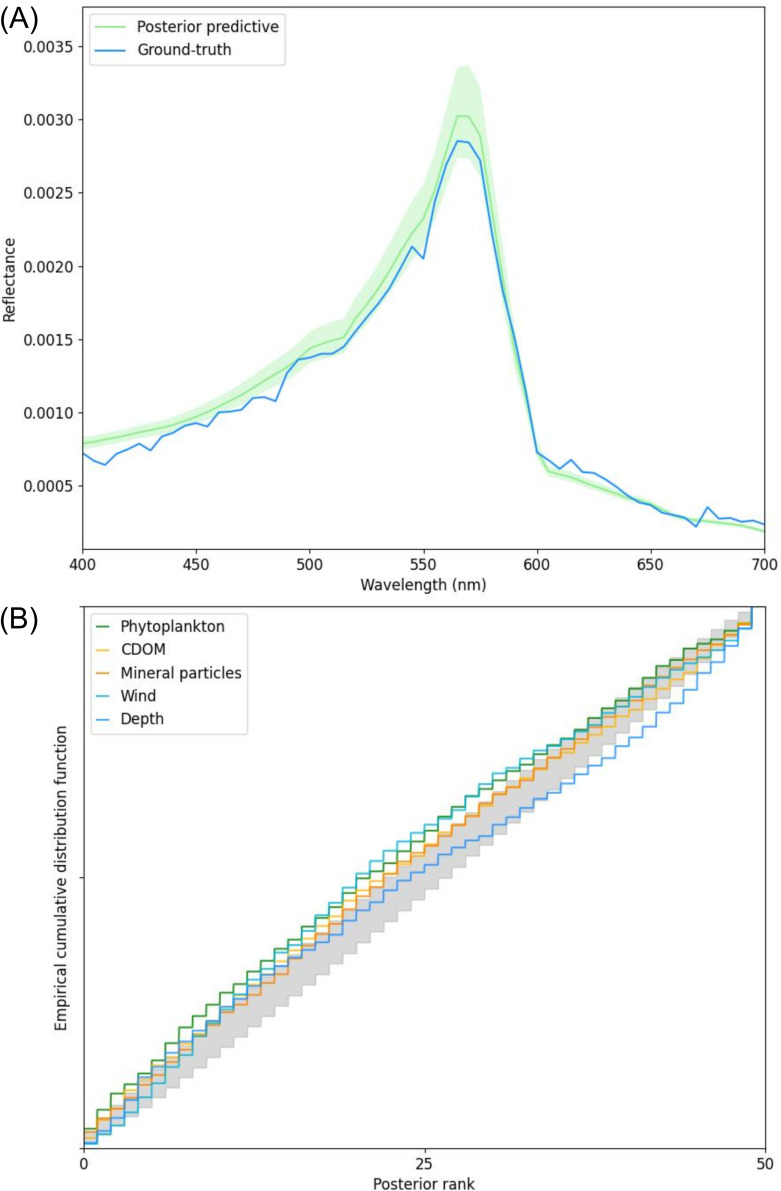
(A) Posterior predictive check. The ground-truth simulated spectrum (blue line) lies within the 5–95 percentiles of the posterior predictive (light green area). The mean of the posterior predictive is shown with the dark green line. (B) The cumulative distribution functions (CDFs) of simulation-based calibration ranks for each of the five θ parameters (each shown with a line of a different colour) with respect to the 95% confidence interval of a uniform distribution (shown in light grey). The plots shown here represent applications of the SBI model trained with hyperspectral spectra characterized by a medium-level signal-to-noise ratio of 100. CDOM = coloured dissolved organic matter.

The inference diagnostics indicated that the SBI algorithm is well calibrated and does not have a systematic bias in posterior estimation. First, the posterior predictive check confirmed that the ground-truth ${\hat{R}}_{rs}$ spectrum is within the 5–95 percentiles of the posterior predictive (figure 5A). Second, simulation-based calibration showed that all the estimated parameters fall within or near the 95% confidence interval of the uniform distribution, evidencing that the posterior mean is well calibrated and is neither systematically under- or over-estimating the parameters, nor is it under- or over-dispersed (figure 5B). Finally, the evaluation of algorithm performance on independently generated simulated data (*n* = 1000) indicated good correspondence between the estimated posterior and the true parameter values. A minimum of 91% coverage probability was achieved for all the inferred parameters, regardless of the signal-to-noise ratio or whether the inference model was trained with hyper- or multi-spectral data (see electronic supplementary material, appendix D for additional results).

### 3.2. Application to field data

**Figure 6.**
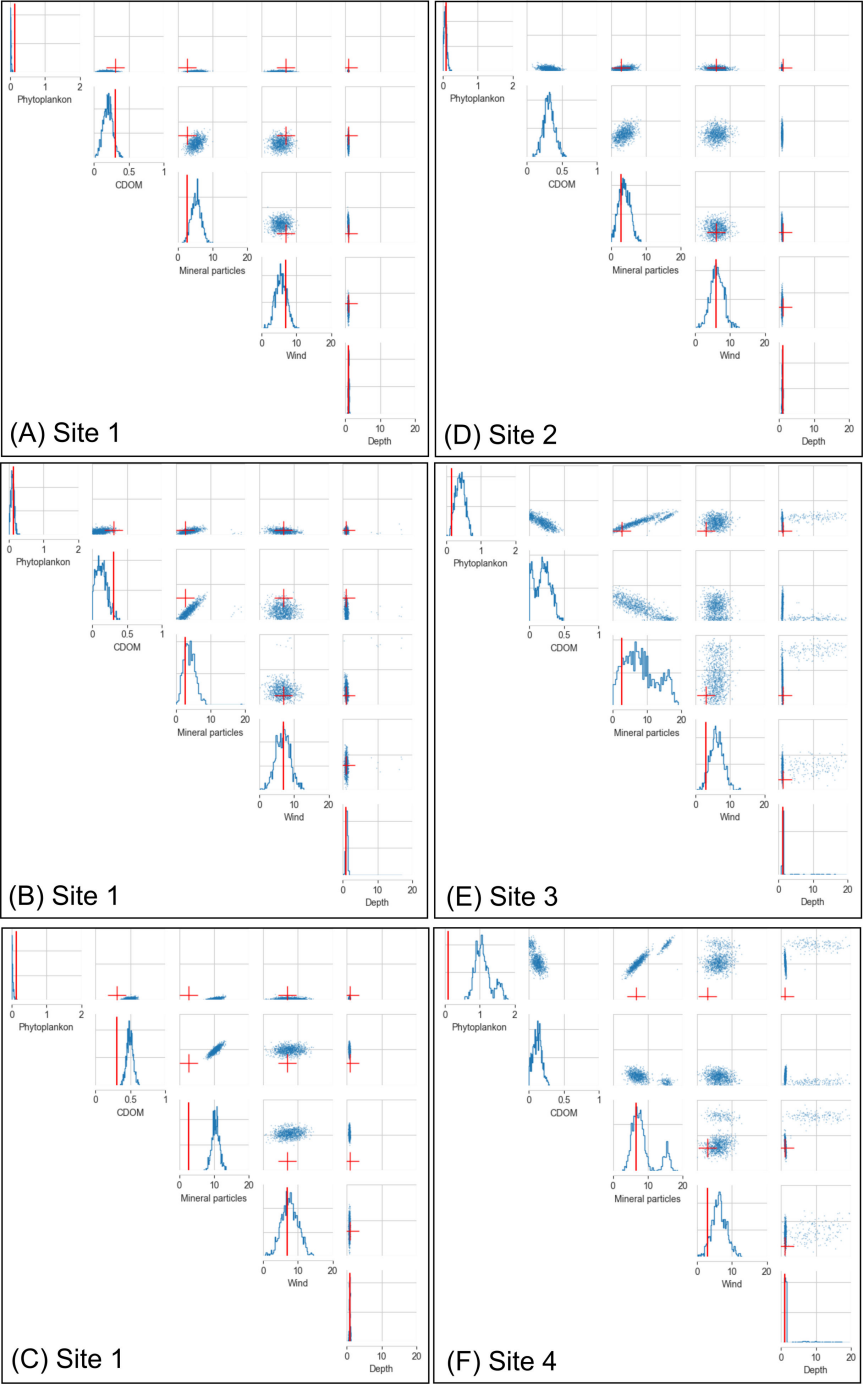
Inference performance on real observations: univariate and pairwise marginalized posterior distributions for the five parameters. (A), (D), (E) and (F) show the results for Sites 1, 2, 3 and 4. These plots were generated with the SBI model trained with hyperspectral data characterized by a signal-to-noise ratio of 100. (B) and (C) show results for Site 1 for the SBI model trained with hyperspectral data characterized by different signal-to-noise ratios 50 and 500, respectively. Coloured dissolved organic matter (CDOM) absorption was only measured at one of the sites (Site 1). The field-measured (ground-truth) values are shown in red, and the posterior distributions are shown in blue. The ground-truth values for minerals concentration are an estimate of suspended particulate matter derived from turbidity data. The suspended particulate matter values shown are the means of the possible ranges (see §2.5.3).

Plots of univariate and pairwise marginalized posterior distributions can be used to visualize the results of statistical inference (figure 6). If the posterior is well calibrated, the true (field-measured) parameter value should lie at a random location (weighted by the posterior mass) within the posterior. The application of the SBI algorithm on the field dataset (*n* = 4) showed that the θ parameters were generally well retrieved (figure 6). Phytoplankton concentration was overestimated at one of the sampling sites (Site 1, figure 6A–C). At Sites 3 and 4, the field-measured values for phytoplankton and wind speed laid at the lower end of the posterior (figure 6E,F).

Out of all inference models (trained with data characterized by different spectral resolutions and noise levels), the inference model trained with hyperspectral data characterized by a medium level of signal-to-noise ratio (100) had the best overall inference performance. Inference performance was assessed based on both coverage probability and the width of the 95% confidence interval calculated from the one-dimensional marginal posterior distribution (electronic supplementary material, appendix D). Coverage probability measures how often the field-measured parameter value falls between the 5th and 95th percentiles of the posterior. The confidence interval width can be used to quantify how confidently each of the parameters is retrieved.

The inference model trained with noisier data (signal-to-noise ratio of 50) produced, in general, slightly wider posterior distributions. In other words, the inference model tended to be less confident about the inferred parameters (figure 6B). The inference model trained with data characterized by the lowest level of noise (signal-to-noise ratio of 500) performed worse, producing posteriors that were not always overlapping with the ground-truth values (figure 6C).

**Figure 7.**
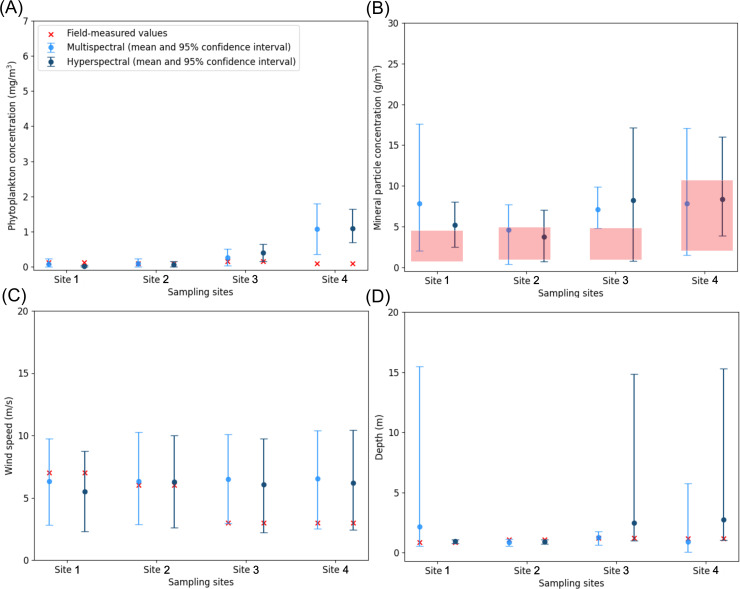
Inference performance on real observations at four sampling sites. The plots shown here apply the SBI model trained with hyperspectral or multispectral data characterized by a medium level of noise (signal-to-noise ratio of 100). (A) phytoplankton concentration, (B) mineral concentration, (C) wind speed and (D) depth. The blue dot corresponds to the posterior mean, while the red cross corresponds to the field-measured (ground-truth) value. The blue bars show the range of the 95% confidence interval associated with the posterior distribution. For minerals concentration (B), the ground-truth values are an estimate of suspended particulate matter derived from turbidity data. To represent the uncertainty associated with the suspended particulate matter estimate, a value range is shown instead of a point estimate (see §2.5.3). Note that the 95% confidence interval does not capture the distribution of the posterior mass within that interval.

**Figure 8.**
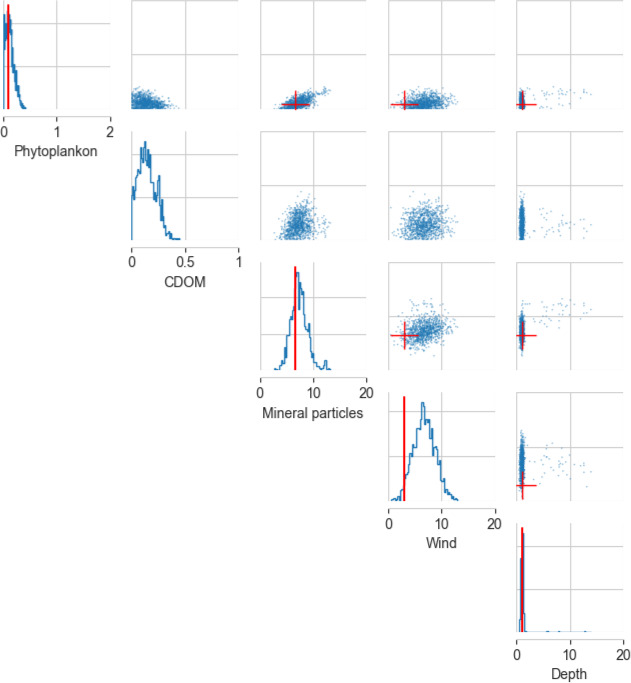
Inference on drone-measured remote-sensing reflectance: univariate and pairwise marginalized posterior distributions for the five parameters. The field-measured (ground-truth) values are shown in red, and the posterior distributions are shown in blue. The ground-truth value for minerals concentration is an estimate of suspended particulate matter derived from turbidity data. The suspended particulate matter value shown is the mean of the possible range (see §2.5.3). No ground-truth measurement of coloured dissolved organic matter (CDOM) is available at this sampling site.

Considering the impact of spectral resolution on inference performance, we found the SBI algorithm to be relatively robust to decreased spectral resolution, with little difference in coverage probability between the hyperspectral and multispectral applications with medium and high levels of noise (figure 7, electronic supplementary material, appendix D). However, decreasing the spectral resolution did make the algorithm somewhat less confident about water constituent estimates, producing slightly wider 95% confidence intervals at most sampling sites (figure 7). Phytoplankton concentration was consistently retrieved with the least uncertainty (narrow posterior distributions) (figure 7). The parameters were accurately retrieved from the drone-measured multispectral remote-sensing reflectance (figure 8).

Note that plotting the 95% confidence interval (figure 7) does not show the distribution of posterior mass within that interval, nor are the potentially multimodal posterior structures visualized. The most meaningful interpretation of the results is obtained by combining insights from figures 6 and 7. For example, when the inference indicated large uncertainty about minerals concentrations (e.g. Sites 3 and 4, inference using hyperspectral data), there was also more uncertainty about depth (figure 7), even though the majority of the posterior mass was still located in a single, narrow peak (figure 6).

## 4. Discussion

The majority of marine remote-sensing algorithms have been developed for optically deep waters [8]. The satellite-based mapping of water constituents in optically shallow environments has received less research attention [25,77,78]. While great advancements have been made in the retrieval of bathymetry and benthic cover type from satellite and drone data [79–82], few studies have focused specifically on developing algorithms for the accurate retrieval of water constituent concentrations in optically shallow environments [77,83]. Importantly, improved inference of optically active water constituents would also help enhance the accuracy of benthic mapping [41,55,84,85].

Here, we have presented an innovative probabilistic machine learning algorithm for the inference of phytoplankton and mineral concentrations, and CDOM absorption from hyperspectral or multispectral data. We found that the likely ranges of water constituent concentrations can be estimated from remote-sensing reflectance in shallow coral reef environments, assuming a single benthic cover. A key advantage of the SBI probabilistic machine learning approach is that the algorithm can be amortized: once trained, it can be applied, under the same assumptions, to new observations without any additional retraining. Applying the amortized algorithm is fast even without graphics processing unit or cloud computing, making SBI a promising approach for large-scale analysis of remotely sensed data. A future water quality mapping workflow could include the delineation of optically shallow/deep waters as a first step [86], and the application of the SBI algorithm tuned for shallow waters as a second step.

Plotting the posterior distributions—the pairwise marginalized posterior distributions in particular—provides useful insights into the likely parameter space corresponding to the observed remote-sensing reflectance. Thereby, the SBI approach provides a new way to address the ill-posed inverse problem of marine remote sensing. Instead of providing a single point estimate as a solution, without knowledge about whether the solution is a global or a local maximum, the SBI approach captures the range of plausible solutions, shown by elongated ellipses in the pairwise marginalized posterior.

Correlation structures in the pairwise marginalized posterior distributions can provide useful insights into the combinatory parameter space that solves the inverse problem. For example, in the field application results of the SBI models trained with datasets characterized by different signal-to-noise ratios, there was a consistent positive relationship in the pairwise marginalized posterior distribution for phytoplankton and minerals. This would suggest that the optical signal (remote-sensing reflectance) would be similar if phytoplankton and mineral concentrations were both either low or high. In this small field dataset, the observed, unintuitive correlation structure may result from small differences in benthic reflectance between sampling sites that the SBI algorithm cannot correctly interpret, as it was trained on a simulated dataset that assumed no variation in benthic reflectance. Nevertheless, the results demonstrate the potential of the SBI approach to offer novel insights into the structure of the parameter space that solves the ill-posed inverse problem. A larger field dataset and the development of approaches to account for the variation in benthic reflectance are required to fully realize the potential of the SBI approach.

Quantifying the widths of the posterior distributions can provide additional insights into the solution of the inverse problem. For example, the SBI algorithm was consistently more confident about phytoplankton concentration compared with the other inferred parameters. This suggests that the signal of phytoplankton pigments can be detected from the spectra even in the presence of the confounding impacts from other optically active constituents, such as suspended minerals. In contrast, the estimation of mineral concentration was often associated with large uncertainty, potentially due to the confounding effect of bottom reflectance.

### 4.1. Limitations and future developments

We evaluated inference performance on a small field dataset. For two out of four sites, field-measured values fell at the lower end of the posterior. Inference performance should be further evaluated on a larger field dataset to confirm whether this results from a small bias in the inference: for a well-calibrated posterior, the field-measured value should lie at a random location within the posterior, weighted by the posterior mass.

Phytoplankton concentration was slightly overestimated at one site. This could be explained by uncertainty associated with the field data due to potential sample degradation before analysis. The IOCCG recommends sample storage in −80°C; however, our field samples were stored at −20°C during transportation to the laboratory [87]. Indeed, field measurements of phytoplankton, suspended minerals and CDOM are characterized by large uncertainties [88,89], with reported average absolute per cent differences of 6–13% between duplicate samples [90,91].

Another source of uncertainty in this first remote-sensing application of the SBI algorithm comes from assuming fixed relations between water constituent concentrations and inherent optical properties [32]. In other words, the inversion is conducted from remote-sensing reflectance directly to biophysical variables (e.g. concentrations of phytoplankton and minerals) rather than inherent optical properties (e.g. absorption and scattering by phytoplankton and minerals) [39,92]. The uncertainty in the bio-optical model is particularly high in shallow coral reef waters, where measurements of water column biophysical variables and their bio-optical counterparts are widely lacking [1,10,93]. The resulting bio-optical model misspecification (i.e. the inability to accurately reproduce the field data) for coral reef waters is a likely explanation for the worse performance of the SBI model when trained on less noisy simulated data. Future work could test implementing the SBI approach to infer inherent optical properties and investigate the uncertainties associated with the bio-optical model conversion between biophysical and optical variables. Additionally, novel approaches for dealing with model misspecification in SBI could be explored [94].

Another interesting avenue for future research would be the investigation of dimensionality reduction of hyperspectral data before inputting it to the conditional density estimator. Reducing the dimensionality of the input data from hyperspectral (61 spectral bands) to multispectral (7 spectral bands) did not drastically decrease inference performance. This suggests that lower-dimensional spectral data may be sufficient for water quality mapping applications. Future work could explore dimensionality reduction, for example using a convolutional neural network that could learn additional informative features from the hyperspectral data, such as spectral shape. Such an approach might be one way to better leverage the wealth of information provided by hyperspectral remote-sensing sensors.

Further field data collection will be needed to thoroughly assess the performance of the SBI algorithm in different environmental conditions, especially in more turbid coral reef waters. Future developments should include variability in benthic reflectance in the simulations and consider the spectral mixing of multiple benthic cover types. Finally, future work could fine-tune the SBI algorithm to specific satellite and drone sensors through the application of sensor-specific, wavelength-dependent noise models.

## 5. Conclusions

The SBI algorithm performance was relatively robust to lower levels of signal-to-noise ratio, although the uncertainty associated with the inferred water constituent concentrations tended to be slightly higher with increased noise. In fact, including too little noise in the simulated dataset resulted in less accurate inference on field data. Uncertainty associated with the bio-optical model may explain why the SBI model trained with the least noisy dataset performed less well; the inference model probably became overly confident about the relations between the biophysical and optical variables.

We identify two research priorities for future work. First, there is a need for further bio-optical data collection to characterize spatio-temporal variability in the optical properties of shallow coral reef waters. A larger field dataset covering a range of environmental conditions is essential for future algorithm development and evaluation. Indeed, we call for a coordinated effort to produce a curated dataset consisting of *in situ* hyperspectral reflectance and water quality measurements from shallow coral reef waters around the world. Second, this first application of SBI to marine remote sensing assumes a single benthic cover (brown coral). Future developments should focus on adapting the algorithm so that it can be applied to satellite pixels that include a mix of different benthic cover types. Establishing a freely available, curated spectral library covering a range of coral species and benthic substrates would help tackle the issue of spectral mixing.

This study advances the mapping of water quality in shallow coral reef environments and applies an innovative probabilistic machine learning approach to address the inverse problem of marine remote sensing. Mapping and monitoring the spatio-temporal dynamics of water constituent concentrations using remote sensing would provide new insights to key ecological and biogeochemical processes taking place in coral reef ecosystems. Ultimately, a better understanding of the variability in water constituent concentrations in coral reef environments could help identify priority sites for local management and restoration action. The SBI algorithm can be used to estimate the likely ranges of phytoplankton and mineral concentrations, absorption by CDOM, wind speed and depth from hyperspectral or multispectral remote-sensing reflectance. This is a significant advantage over traditional spectral optimization methods that only provide a single solution without an estimate of confidence in the result. Another important advantage of our approach is that the SBI algorithm is amortized: once it has been trained on simulated data, it can be applied to new observations without retraining. This makes SBI a promising approach for computationally efficient analysis of large amounts of satellite and drone data.

## Data Availability

All data and code used in the study are open source and freely available via the Open Science Framework data repository [49] and the associated GitHub repository [95]. Supplementary material is available online [96].
